# Effects of Cash Transfer and Incentive Programs on Service Utilization and Treatment Outcomes Related to Neglected Tropical Diseases and Their Impact on Health and Nutrition in Low- and Middle-Income Countries: Protocol for a Systematic Review

**DOI:** 10.2196/76450

**Published:** 2026-03-19

**Authors:** S M Tafsir Hasan, Radhika Dayal, Amena Al Nishan, Sunetra Ghatak, Puja Chakraborty, Sudeshna Maitra, Sumaiya Tasneem Raisa, Nizamuddin Khan, Dinesh Mondal, Tahmeed Ahmed, Avishek Hazra

**Affiliations:** 1 International Centre for Diarrhoeal Disease Research Bangladesh (icddr,b), 68, Shaheed Tajuddin Ahmed Sarani, Mohakhali, Dhaka, 1212, Bangladesh, 880 1709651470; 2 PopulationCouncil Consulting Private Limited, Noida, Uttar Pradesh, India; 3 O. P. Jindal Global University, Sonipat, Haryana, India

**Keywords:** neglected tropical disease, cash transfer, incentive program, service utilization, treatment outcome, nutrition, health, low- and middle-income countries

## Abstract

**Background:**

Despite a global decreasing trend in neglected tropical diseases (NTDs), progress is not on track to meet the Sustainable Development Goals by 2030. Evidence suggests a positive effect of conditional cash transfer programs on controlling NTDs. However, it remains to be evaluated whether other financial incentive programs exert similar effects on NTD-related service utilization and treatment outcomes in low- and middle-income countries (LMICs).

**Objective:**

This systematic review aims to evaluate the effects of cash transfer and incentive programs on NTD-related service utilization and treatment outcomes in LMICs; to examine how covariates, program design, and implementation plan influence these outcomes; and to examine how NTD-related service utilization and treatment outcomes influence nutritional status and health in LMICs.

**Methods:**

This review will follow the PICOS (Population, Interventions, Comparators, Outcomes, Study Design) framework. The population of this review will be restricted to adults and children of all ages in LMICs. For intervention selection, we will include any program or policies addressing socioeconomic disadvantage through the provision of cash transfers or incentive programs, including but not limited to in-kind transfers, food vouchers, free medicine vouchers, discount coupons, and microcredit to households or individuals. Eligible comparators will be the population who did not receive any intervention, recipients of interventions from before the intervention, and populations or areas exposed to different levels of intervention coverage. The primary outcomes will be service utilization and treatment outcomes related to any of the 21 NTDs prioritized by the World Health Organization. Studies without any financial incentive intervention will be excluded. Studies available in English between 2000 and 2024 will be searched in databases, including PubMed, Google Scholar, Scopus, Cochrane, Embase, and CINAHL. Screening, data extraction, and risk of bias assessment will be conducted independently by two reviewers, and discrepancies, if any, would be resolved by a third reviewer. Quality assessment will use the Revised Cochrane Risk of Bias for randomized trials (RoB 2), Risk of Bias in Non-Randomized Studies of Interventions (ROBINS-I), the Joanna Briggs Institute (JBI) checklist for cross-sectional studies, and the Critical Appraisal Skills Programme (CASP) tool for qualitative studies. Data synthesis will involve narrative summaries by tables and figures, a meta-analysis will be undertaken, and subgroup analyses will be performed where feasible.

**Results:**

This project is supported by the Children’s Investment Fund Foundation and was funded in March 2024. As of January 15, 2026, article search and title-abstract screening have been completed, and full-text screening is expected to be completed by February 2026. Findings will then be synthesized and reported by the end of April 2026.

**Conclusions:**

The limitations of the study may include potential language bias and methodological heterogeneity, which may limit the feasibility of meta-analysis. The findings of this review will inform policymakers in developing more effective strategic plans for controlling NTDs in LMICs.

## Introduction

### Background

Neglected tropical diseases (NTDs) remain a major global public health concern, affecting an estimated 1.62 billion people in tropical and subtropical climatic zones [1,2] and accounting for approximately 200,000 deaths and 14.5 million disability-adjusted life years annually [2,3]. Although there has been a general decline in the global burden of NTDs [2], the progress is not yet on track to meet the ambitious Sustainable Development Goals 2030 target of a 90% reduction in the number of people requiring interventions for NTDs [3]. NTDs continue to pose a big challenge for low- and middle-income countries (LMICs) due to their intricate links with poverty, inadequate hygiene, poor sanitation, and limited health care access [4,5].

Poverty is a key factor exacerbating the burden of NTDs in LMICs. Financial deprivation and disease are closely intertwined, which influence the social and environmental determinants of NTDs, including water, sanitation, and hygiene, education, housing, and health care accessibility [5-7]. Poverty highly modulates the accessibility and utilization of the services related to the 5 important NTD control strategies recommended by the World Health Organization (WHO): innovative and intensified disease management; preventive chemotherapy; vector control; veterinary public health measures; and provision of safe water, sanitation, and hygiene practices [8,9]. NTDs disproportionately affect impoverished and marginalized communities, resulting in increased catastrophic health spending and out-of-pocket expenses [10]. Already living in poverty before the onset of these diseases, affected households often face deeper financial hardship as rising out-of-pocket costs push them further below the poverty line [11-13]. To manage these expenses, they often resort to coping strategies such as borrowing money or selling assets, which further exacerbates their poverty. This cycle undermines access to health care services and negatively affects treatment outcomes [13]. Physical disabilities due to NTDs, such as blindness, lesions, and swelling, may also contribute to decreased income ability [14]. This financial burden perpetuates a cycle of poverty and NTD burden, hindering service uptake and treatment [15]. Consequently, the detrimental effects of NTDs are amplified, including poor NTD-related service utilization and adherence, treatment failure, disability, and deterioration of general health and well-being [15].

Moreover, infection with NTDs increases the nutritional burden of the hosts and impedes overall health. NTDs have been reported to be associated with stunting, anemia, iron deficiency, micronutrient deficiencies, and malnutrition [16-19]. Simultaneously, nutrient deficiency may also increase the susceptibility and vulnerability of NTD-affected individuals and lead to NTD treatment failure [18,20,21]. Actions taken to control NTDs may eventually improve nutritional outcomes, which, in turn, will also influence overall health and well-being, particularly in resource-limited settings.

Despite various programs and policies implementing innovative strategies to improve NTD campaign coverage, multiple layers of poverty-associated and financial obstacles continue to hinder appropriate health-seeking behaviors in affected populations. These barriers include attending screening clinics, receiving adequate care, and treatment adherence [22-25]. Cash transfer programs have demonstrated potential for improving NTD outcomes [26-29]. These programs aim to provide small-scale monetary assistance to individuals or households to break the intergenerational cycle of poverty. Conditional cash transfers (CCTs) impart benefits through a 2-way strategy: direct cash assistance and enforced conditionalities [30]. It is also possible that conditionalities related to the control of NTDs may also impart positive effects on nutrition and overall health outcomes in LMICs. Large-scale, national-level cash transfer programs in Latin America have not only proven effective in improving social protection, economic stability, and sustained developmental outcomes [31] but have also generated interest in using cash transfers to target context-specific health outcomes, including NTD treatment outcomes, maternal health, child health, cognitive development, nutrition, and health care utilization [26,32-34].

A recent systematic review highlighted the positive impact of CCT programs in controlling NTDs. It indicated that the targeted benefits of these programs for vulnerable populations can help reduce health inequalities associated with NTDs. For example, cash transfers have been shown to reduce leprosy incidence and increase the uptake of deworming treatments in certain contexts [35]. However, the review had a major limitation in that it included and synthesized evidence for only 3 of the 21 NTDs and disease groups prioritized by WHO [2,3,35]. While other incentive programs, such as in-kind transfers, microcredits, vouchers for transport, medicines, or food, have shown the potential to promote health knowledge, reduce health inequalities, and improve health-seeking behaviors, their direct or indirect effects on NTD-related indicators have not been comprehensively studied in LMICs [36,37].

Given this context, there is a need for an evidence synthesis on the effects of cash transfers and other incentive programs on NTD-related service utilization and treatment outcomes in LMICs. This proposed systematic review and meta-analysis (depending on information availability) will conduct a comprehensive synthesis and unbiased assessment of the existing studies in this area, critically assessing the strengths and limitations of existing evidence.

### Objectives

The systematic review aims to answer the following three questions: (1) What are the effects of cash transfer and incentive programs on NTD-related service utilization and treatment outcomes in LMICs? (2) How do incentive program design (type, amount, and frequency), implementation plan, and other covariates influence NTD-related service utilization and treatment outcomes in LMICs? and (3) How do NTD-related service utilization and treatment outcomes influence nutritional status, overall health, and well-being in LMICs?

## Methods

### Overview

This protocol was developed in accordance with the guidelines of the PRISMA-P (Preferred Reporting Items for Systematic Review and Meta-Analysis Protocols) [38]. A PRISMA-P checklist for this protocol is provided in Checklist 1. The protocol has been registered in PROSPERO (CRD42024627804).

### Eligibility Criteria

The eligibility criteria for studies to be included in this review are adapted based on the PICOS (Population, Interventions, Comparators, Outcomes, Study Design) framework [39], as described below.

#### Population

The population of interest in this review will be adults and children of all ages residing in LMICs.

#### Interventions

For intervention, this review will include programs or policies addressing socioeconomic disadvantages through the provision of cash transfers or financial incentive programs, including but not limited to in-kind transfers, food vouchers, free medicine vouchers, discount coupons, and microcredit to households or individuals.

CCT programs require beneficiaries to comply with certain conditionalities (eg, regular health check-ups) [31], while unconditional cash transfer programs do not set such requirements [40]. Microcredit is the extension of very small loans (microloans) to impoverished borrowers who typically lack collateral, steady employment, and a verifiable credit history [41]. It is designed to support entrepreneurship and alleviate poverty [41]. An in-kind transfer is a transfer of goods or services rather than cash [42]. Other incentive programs may also include giving free medicine vouchers, food vouchers, discount coupons, social protection, social programs, scholarship programs, family allowances, and microfinance.

#### Comparators

This review will include studies with suitable comparators; studies without comparators will be excluded. Comparators will include individuals or households who did not receive any cash transfers or incentive programs, recipients of cash transfers or incentive programs from the preintervention period, and populations or areas with varying levels of intervention coverage.

#### Outcomes

The primary outcomes of interest will be service utilization and treatment outcomes related to any of the 21 NTDs and disease groups prioritized by WHO [2]. These include Buruli ulcer; Chagas disease; dengue and chikungunya; dracunculiasis; echinococcosis; foodborne trematodiases; human African trypanosomiasis; leishmaniasis; leprosy; lymphatic filariasis; mycetoma, chromoblastomycosis, and other deep mycoses; noma; onchocerciasis; rabies; scabies and other ectoparasitoses; schistosomiasis; soil-transmitted helminthiases; snakebite envenoming; taeniasis/cysticercosis; trachoma; and yaws.

Service utilization related to WHO-recommended core strategic interventions for each of the 21 NTDs and disease groups will be evaluated. The aspects of service utilization evaluated will include, but not be limited to, health-seeking behavior, attendance at screening clinics, treatment adherence, and NTD campaign coverage. Treatment outcomes related to WHO-recommended core strategic interventions for each of the 21 NTDs will include, but not be limited to, prevalence, incidence, detection rate, cure rate, disability rate, death rate, and components of morbidity management, including surgery, wound management, physiotherapy, and long-term rehabilitation. A comprehensive list of core strategic interventions and treatment outcomes for each of the 21 NTDs and disease groups is provided in Table 1 [1,3,43-48].

**Table 1.** List of core strategic interventions and treatment outcomes for each of the 21 neglected tropical diseases (NTDs) and disease groups.

Serial	NTD name	Core strategic interventions					Treatment outcomes
		Innovative and intensified disease management	Preventive chemotherapy	Vector control	Veterinary public health measures	Provision of safe WASH^a^ practices	
1	Buruli ulcer	Early detection, case management (provision of antibiotics: rifampicin, clarithromycin, moxifloxacin, streptomycin, and hygienic wound care)	Bacillus Calmette–Guérin (BCG) vaccination	Aquatic insects, adult mosquitoes, possums, and biting arthropods	Not applicable	Safe water storage practices	Disability, morbidity management including surgery, wound, and lymphedema management, physiotherapy and long-term rehabilitation, prevalence, incidence, detection rate, cure rate, disability rate, death rate
2	Chagas disease	Case management (benznidazole and nifurtimox), MDA^b^, surgical treatment	Blood screening before blood transfusion and organ transplantation	Spraying with residual insecticides to remove triatomine bugs, home cleanliness, and housing improvements (eg, crack-free walls, bed nets), wear protective clothing	Not applicable	Good hygiene practices	Prevalence, incidence, detection rate, cure rate, surgery rate, disability rate, death rate
3	Dengue and chikungunya	Case management (fluid management, proactive treatment of hemorrhage, such as platelet transfusion or whole blood transfusion, paracetamol)	Vaccination (dengue)	Reducing mosquito habitats (eg, environmental modification, use of lids), wire mesh, mosquito net, mosquito trap, mosquito insecticide, mosquito repellents	Not applicable	Safe water storage practices, safe water disposal, waste management	Prevalence, incidence, detection rate, cure rate, disability rate, death rate
4	Dracunculiasis	Surveillance, early detection of cases, case containment, cash reward scheme for voluntary reporting, metronidazole or thiabendazole, health education	Not applicable	Temephos to control water fleas/cyclops	Proactive tethering of dogs	Safe drinking water supply and sanitation, water filtration, preventing water contamination	Prevalence, incidence, detection rate, cure rate, disability rate, death rate
5	Echinococcosis	Albendazole prophylaxis, surgery, early detection by ultrasound	Not applicable	Not applicable	Deworming of dogs, foxes, vaccination of sheep, safe slaughtering and disposal	Handwash, avoiding contaminated food and water	Prevalence, incidence, detection rate, cure rate, disability rate, surgery rate, total or partial cystopericystectomy, liver damage, death rate
6	Foodborne trematodiases	Anthelminthic medicines (praziquantel, triclabendazole), PAIR (puncture, aspiration, instillation, and respiration) methods, surgery (partial hepatectomy), Provision of care and rehabilitative services	MDA with triclabendazole (*Fasciola* spp.) and praziquantel (small liver flukes and *Paragonimus* spp.)	Not applicable	Livestock and domestic animal treatment, safe fish farming, one health intervention	Safe disposal of fecal waste, safe water, safe food practices, safe WASH practices	Prevalence, incidence, detection rate, cure rate, disability rate, surgery rate, liver damage, death rate
7	Human African trypanosomiasis	Screening clinic attendance, active case detection, treatment of gHAT^c^: pentamidine, eflornithine and nifurtimox, fexinidazole, melarsoprol, suramin	Not applicable	Baits and traps, impregnated screens, insecticide spraying, reducing the disease reservoir, controlling the tsetse fly vector	Treatment of animals (cattle, pigs)	Safe water, safe WASH practices	Prevalence, incidence, detection rate, cure rate, disability rate, death rate
8	Leishmaniasis	Early diagnosis, cryotherapy, thermotherapy, medication (liposomal amphotericin B, pentavalent antimonials, miltefosine), rapid diagnostic test rk-39	Not applicable	Insecticide spraying, insecticide-treated nets, environmental management, protection from sandfly bites	Rodent control, vaccination for dogs	Safe WASH practices	Prevalence, incidence, detection rate, cure rate, disability rate, death rate
9	Leprosy	Early detection, MDA, physical therapy, multidrug therapy (rifampicin, clofazimine, dapsone, ofloxacin, and minocycline)	Postexposure prophylaxis of rifampicin, vaccination with BCG, Mycobacterium w (Mw)	Not applicable	Not applicable	WASH in health facility	Prevalence, incidence, detection rate, cure rate, disability rate, death rate
10	Lymphatic filariasis	Essential package of care (skincare and hygiene, exercises for lymphedema, treatment of adenolymphangitis), surgery to cure hydrocele, antifilarial medicines (albendazole, diethylcarbamazine citrate), ivermectin for lymphatic filariasis co-endemic countries	MDA, annual MDA with diethylcarbamazine citrate and albendazole, home-based disability alleviation and prevention	Vector control to reduce transmission, avoid mosquito bites	Not applicable	Hygiene, sanitation improvement, personal hygiene and self-care of the affected limbs to avoid secondary infections, agriculture, livestock, wildlife, environment (One Health), avoid mosquito bites	Prevalence, incidence, detection rate, cure rate, disability rate, hydrocele management, surgery rate, mental health, death rate
11	Mycetoma, chromoblastomycosis and other deep mycoses (paracoccidioidomycosis [PCM], caused by *Paracoccidioides* spp. and sporotrichosis [ST], caused by *Sporothrix* spp.)	Long-term itraconazole+ antibiotic administration, physical therapy, wound care, surgery (excision, debridement, amputation)	Self-protection by wearing long clothes, closed-toed shoes	Not applicable	Pet control for sporotrichosis, feral cat management	Personal hygiene and self-care of the affected limbs to avoid secondary infections	Prevalence, incidence, detection rate, cure rate, disability rate, local excision, debridement, amputation, surgery rate, death rate
12	Noma	Case management with antibiotics, mouthwash, health education	Nutrition supplements	Not applicable	Not applicable	Personal hygiene	Prevalence, incidence, detection rate, cure rate, disability rate (disfiguration), mental health, death rate
13	Onchocerciasis	Ivermectin, doxycycline, eye care, MDA	MDA of ivermectin for community-based prevention	Insecticides at black fly habitats and larval breeding sites, avoid blackfly bites	Not applicable	Not applicable	Prevalence, incidence, detection rate, cure rate, disability rate (ocular morbidity rate), death rate
14	Rabies	Postexposure prophylaxis (PEP) with rabies vaccine and rabies immunoglobulin	Pre-exposure vaccination for people at high risk (eg, laboratory staff working with rabies virus, veterinarians and animal handlers)	Wild mammals (dogs, bats) control	Mass vaccination of dogs, mammal pet vaccination, agriculture, livestock, wildlife, environment (One Health)	Not applicable	Prevalence, incidence, detection rate, death rate
15	Scabies and other ectoparasitoses	Topical scabicides, permethrin, benzyl benzoate, malathion, and sulfur ointment, oral ivermectin	MDA using oral ivermectin	Not applicable	Not applicable	WASH practices, hygiene practices	Prevalence, incidence, detection rate, cure rate, co-morbidities (kidney disease, rheumatic heart disease, systemic infection)
16	Schistosomiasis	Case management and surgery, education	MDA with praziquantel	Snail control	Keeping animals away from transmission sites	Safe water, improved sanitation, excreta management, hygiene	Prevalence, incidence, detection rate, cure rate, disability rate, surgery rate, death rate
17	Soil-transmitted helminthiases	Case management (with albendazole/mebendazole/ivermectin), education of hygiene	MDA with albendazole/mebendazole/ivermectin, antenatal care preventive treatment	Not applicable	Not applicable	WASH practices, safe water, improved sanitation, excreta management, hygiene	Prevalence, incidence, detection rate, cure rate, disability rate, death rate
18	Snakebite envenoming	High-quality, safe, and effective snake antivenoms; wound care; ventilation; surgery; WHO^d^ Regional Action Plan for prevention and control of snakebite envenoming 2022‐2030	Protective footwear, sealing house wall, roof and door gaps, bed nets, household rodent controls to keep away the snakes	Not applicable	Not applicable	Not applicable	Prevalence, incidence, detection rate, cure rate, disability rate, surgery rate, intensive care unit (ICU) admission rate, death rate
19	Taeniasis/cysticercosis	Case management (praziquantel, niclosamide, albendazole), neurological treatment, surgery	MDA with niclosamide/ praziquantel/ albendazole, community awareness	Not applicable	Improved pig husbandry, pig vaccination	WASH practices, proper cooking	Prevalence, incidence, detection rate, cure rate, disability rate (neurological symptoms), surgery rate, death rate
20	Trachoma	Eye surgery to treat trachomatous trichiasis, TT (the S of the SAFE strategy) Azithromycin	MDA of azithromycin and tetracycline eye ointment (WHO-recommended SAFE strategy)	Reducing breeding sites for muscid flies	Not applicable	Access to water and improved sanitation	Prevalence, incidence, detection rate, cure rate, disability rate (blindness), surgery rate
21	Yaws	Case management (azithromycin medication, intramuscular benzathine benzylpenicillin), Morges strategy, health education	Mass treatment, also known as total community treatment (TCT), involves giving oral azithromycin (single dose)	Not applicable	Not applicable	Personal hygiene	Prevalence, incidence, detection rate, cure rate, disability rate (disfiguration)

a WASH: water, sanitation, and hygiene.

b MDA: mass drug administration.

c gHAT: gambiense human African trypanosomiasis.

d WHO: World Health Organization.

The review will include only studies reporting primary outcomes. However, in the included studies, exploratory outcomes related to the nutrition, health, and well-being of adults and children will be analyzed to examine how NTD-related service utilization and treatment outcomes may influence these outcomes in LMICs. Exploratory outcomes will include, but not be limited to, nutritional status, various types of malnutrition, micro- and macronutrient deficiency, anemia, cognitive development, school attendance and performance, mortality, and morbidity.

#### Study Design

The review will include randomized controlled trials, as well as nonrandomized studies with comparator groups, such as cohort, case-control, longitudinal, cross-sectional, ecological, and quasi-experimental studies. Additionally, impact evaluation results, program reports, qualitative studies, and studies employing methodologies like difference-in-difference, pre-post design, interrupted time series, regression discontinuity, instrumental variable estimation, and propensity score matching will be considered. Studies reported in the English language will be considered for inclusion.

### Search Strategy

A scoping search will be conducted to identify key Medical Subject Headings terms and other relevant search terms, which will then be combined to develop a comprehensive search strategy. Boolean operators (AND, OR, and NOT), truncation/stemming (*), and phrase searching (“”) will be used. The search will focus on NTDs, using genus names along with key terms, such as cash transfer, unconditional cash transfer, CCT, microcredit, food voucher, and incentive. A preliminary search strategy is provided in Table 2.

**Table 2.** Preliminary search strategy.

Domain	Description	Search terms or keywords
Population	Participants	Adults and children living in LMICs^a^ Adults, young adults, young women, young men, child, infant, adolescents, teen
Interventions	Programs	Money, monetary, cash, financial transfer, cash transfer, payment, conditional cash transfer (CCT), unconditional cash transfer (UCT), incentive. Financial, nonfinancial incentive programs, eg, social protection, social program, scholarship programs, family allowance, in-kind transfer, food/medicine voucher, and microfinance/microcredit.
Outcomes	Neglected tropical diseases and disease groups; core strategic interventions; NTD^b^-related service utilization; and treatment outcomes	Neglected disease, neglected tropical disease, tropical medicine, tropical disease, Buruli, buruli ulcer, Bairnsdale ulcer, Daintree ulcer, Mossman ulcer, mycobacterium, *Mycobacterium ulcerans*, Chagas, Chagas disease, trypanosome, *Trypanosoma cruzi*, American trypanosomiasis, dengue, dengue virus, break-bone disease, break-bone fever, chikungunya, dracuncul, dracunculus nematode, dracunculiasis, guinea worm, guinea-worm, antihelminthic, antiparasitic, deworm, *Echinococcus*, echinococc, foodborne, trematodiases, trematodias, clonorchiasis, opisthorchiasis, fascioliasis, paragonimiasis, human African trypanosomiasis, sleeping sickness, Leishmaniasis, kala-azar, leprosy, Hansen, trypanosome, lymphatic filariasis, filaria, elephantiasis, *Wuchereria bancrofti*, brugia, onchocerciasis, rabies, river blindness, schistosome, bilharzia, helminth, roundworm, nematode, soil transmitted helminthiasis, ascaris, whipworm, trichuris, hookworm, ancylostom, taenia, cysticercos, chlamydia, tapeworm, hookworm, *Necator americanus*, *Ancylostoma duodenale*, taenia, cysticercosis, cestod, trachoma, treponem, noma, snake bite envenoming, yaws, frambesia, bejel, pinta, parangi, mycetoma, chloroblastomysis, chromomyc, mycosis, mycos, scabies, ectoparasite, mosquito habitat, Temephos, water fleas, small liver flukes, fecal waste, feces, lymphedema, adenolymphangitis, hydrocele, secondary infection, agriculture, wildlife, environment, sporotrichos, feral cats, black fly habitat, larval breeding, wild mammals, veterinarians, animal handlers, rabies virus, pig husbandry, breeding sites, etc Early detection, antibiotic, Rifampicin, Clarithromycin, hygiene, wound care, BCG^c^ vaccine, Benznidazole, Nifurtimox, MDA^d^, mass drug administration, surgery, blood screening, blood transfusion, organ transplantation, insecticide, bed nets, spraying, fluid management, wire mesh, repellents, safe water practices, waste management, surveillance, cash reward scheme, voluntary reporting, Cyclops, dogs, water filtration, Albendazole, early detection by USG, deworming, deworming of dogs, sheep vaccination, handwash practices, anthelminthic medicines, Praziquantel, PAIR^e^ methods, partial hepatectomy, rehabilitation, Triclabendazole, Praziquantel, one health, WASH^i^ practices, screening, Pentamidine, Eflornithine, Fexinidazole, baits, cryotherapy, thermotherapy, liposomal Amphotericin B, pentavalent antimonials, Miltefosine, rodent control, dog vaccination, physical therapy, postexposure prophylaxis, PEP^f^, anti-filarial medicines, vector control, sanitation, limb care, excision, debridement, amputation, long clothes, closed-toed shoes, pet control, mouthwash, nutrition, nutrition supplement, Ivermectin, Doxycycline, eye care, pre-exposure vaccination, scabicides, snail control, antenatal care, ANC, excreta management, antivenom, ventilation, footwear, Niclosamide, neurological treatment, pig vaccination, eye surgery, Azithromycin, Tetracycline, eye ointment, SAFE strategy, muscid flies, personal hygiene. Attendance at screening clinics, treatment adherence, health-seeking behavior, NTD campaign coverage, etc Prevalence, incidence, detection rate, cure rate, disability, disability rate, morbidity, morbidity rate, death rate, lymphedema management, wound management, total cystopericystectomy, partial cystopericystectomy, surgery rate, liver damage, mental health, disfiguration, ocular morbidity, comorbidities, kidney disease, rheumatic heart disease, systemic infection, ICU^g^, intensive care unit, admission rate, blindness,
Study	Design	RCTs^h^, nonrandomized studies, cohort studies, longitudinal studies, case-control studies, cross-sectional studies, ecological studies, quasi-experimental studies, impact evaluation results, and program reports. Difference-in-difference, pre-post design, time series, regression discontinuity, instrumental variable estimation, propensity score matching, and qualitative study.

a LMICs: low- and middle-income countries.

b NTD: neglected tropical diseases.

c BCG: Bacillus Calmette–Guérin.

d MDA: mass drug administration.

e PAIR: puncture, aspiration, injection, and re-aspiration.

f PEP: postexposure prophylaxis.

g ICU: intensive care unit.

h RCT: randomized controlled trial.

i WASH: water, sanitation, and hygiene.

### Article Screening Process

The following databases will be searched: MEDLINE, PubMed, Scopus, Embase, Web of Science, POPLINE, Google Scholar, Cochrane Collection, PsycINFO, Global Health, EconLit, Social Sciences Citation Index, International Bibliography of the Social Sciences, Knowledge Commons of Population Council, and 3ie database. Gray literature databases, including WHO websites, AlignMNH, World Bank, and UNICEF websites, will also be searched for relevant reports. Websites and online resources of universities and research centers in LMICs will be reviewed to identify working papers, dissertations, and theses. Reference lists or bibliographies of included studies will also be hand-searched to include any additional relevant studies. For those studies not available in the public domain, a librarian will be contacted. Studies in English, published or available from 2000 to 2024, will be included. A search log will be maintained throughout the search process to document the database platforms or search interface used, the date the search was conducted, the search strings used, and the number of records or counts identified. The log will be updated as necessary to ensure transparency, robustness, and ease of updating during the study period.

Preliminary search documents will be stored in reference management software (EndNote or Mendeley). All the screened studies will be transferred into Microsoft Excel or Rayyan tool, a systematic review management platform [49].

### Title, Abstract, and Full-Text Relevancy

Two reviewers will independently screen studies based on titles and abstracts. During the initial screening phase, decisions made by each reviewer will be blinded. After the initial screening is done, the studies screened by the two reviewers will be compared and checked for discrepancies. Any discrepancies in the categorization will be resolved through consultation between the reviewers or with another reviewer. A second-level independent review will then be conducted by a separate reviewer to perform quality checks on studies with discrepancies and those deemed ineligible. Studies deemed relevant based on titles and abstracts will undergo full-text screening for relevancy, following the same procedures used during the title and abstract screening phase. Reasons for the exclusion of papers will be documented. The studies will be equally divided among the reviewers for relevancy screening. Data will be extracted individually by each reviewer. Any questions arising during the process will be addressed through consultation among the reviewers to reach a consensus. Eligible studies from the full-text review will be entered directly into a master data extraction spreadsheet.

### Data Extraction

After the article screening process is completed, all eligible studies will be extracted into a master data extraction spreadsheet. The data extraction sheet will include the following categories: title, author(s), date of publication, type of publication (eg, summary, synthesis, single study), number and type of included studies (if a summary or synthesis), settings and population studied, interventions implemented, outcomes measured and results, and if relevant, whether results differed among subgroups, such as gender, socioeconomic status, or ethnicity. For studies with multiple outcomes or variables, all eligible outcomes will be extracted. The data extraction form will be pilot-tested and refined based on the information extracted from a few initial studies meeting the inclusion criteria. This process will help minimize bias and improve the validity and reliability of the systematic review. Authors will be contacted through email if key information is missing or if further information or clarification is required.

### Risk of Bias Assessment for Individual Studies

The quality assessment will be done by using the Revised Cochrane Risk of Bias for randomized trials (RoB 2) [50], the Risk of Bias in Non-Randomized Studies of Interventions (ROBINS-I) [51], the Joanna Briggs Institute (JBI) checklist for analytical cross-sectional studies, and an adapted version of the Critical Appraisal Skills Programme (CASP) [52] for qualitative studies; ecological studies will be evaluated narratively. The quality of each included study will be independently assessed by one reviewer, with the assessment results checked by a second reviewer. Any discrepancies or disagreements will be resolved through consultation with a third reviewer to reach a consensus.

### Anticipated Number of Studies to Be Included in the Review

Based on the initial search to develop the protocol, about 20 to 30 studies are anticipated to be included in the review [34,53,54]. This number might change during the actual work of the systematic review based on the search using electronic databases and gray literature.

### Data Synthesis

A narrative synthesis will be produced by describing the studies. A geographical information system map will be generated to illustrate the frequency distribution of impact evaluations of cash transfer and incentive programs on NTD-related service utilization, treatment outcomes, nutritional status, and health and well-being, subject to data availability. Interventions of cash transfers and incentive programs will be classified into different categories for different types of NTDs, and their effects will be synthesized for (1) NTD-related service utilization; (2) treatment outcomes; and (3) nutritional status, health, and well-being outcomes. For this review, improvement in nutritional status and health and well-being are considered potential downstream or contextual outcomes that may occur through multiple pathways, in addition to through changes in NTD-related service utilization and treatment outcomes. These pathways may include independent effects of incentives on household resources, food security, health-seeking behavior, and overall living conditions. Given that most primary studies may not be designed to test the full causal pathway, causal inference in this review will be restricted to proximal outcomes (service utilization and treatment outcomes), while nutritional and broader health outcomes will be interpreted as associative and contextual. All the studies will be tabulated by quantitative and qualitative type, and key findings will be described. Primary and exploratory outcomes will be listed along with differences in effectiveness between different cash transfer and incentive programs for NTD control.

We will assess the certainty of evidence for quantitative outcomes using the GRADE (Grading of Recommendations, Assessment, Development and Evaluation) approach. For qualitative or narrative evidence, we will use an appropriate structured approach (eg, GRADE-CERQual) to evaluate confidence in the findings.

The possibility of performing a meta-analysis will be explored depending on the availability of a sufficient number of relevant studies. The ability to undertake a meta-analysis will depend on the heterogeneity of the measures, such as similarities or differences in population, intervention, outcomes, and methodologies. If a meta-analysis is deemed appropriate, statistical heterogeneity of effects will be assessed using *χ*^2^ and *I*^2^ statistics. Efforts will be made to analyze the factors explaining heterogeneity through moderator analysis, including subgroup meta-analysis. All analyses will be contingent on sufficient study density.

For qualitative studies, the themes will be identified using a deductive approach, and the findings will be used to interpret or complement the quantitative findings as part of the narrative synthesis. We will also consider stratifying synthesis by study design strength.

The synthesis will present the factors such as program design, implementation, context, and challenges that influence the impact of cash transfer or incentive programs on NTD-related service utilization, treatment outcomes, nutrition status, and health and well-being. Absolute evidence gaps and synthesis gaps will also be identified that can inform the development of new studies or programs.

### Analysis of Subgroups or Subsets

The possibility of performing subgroup analyses will be explored. It will, however, depend on the evidence strength to summarize effects in special populations, such as pregnant women and geriatric populations, or to distinguish effect differences across different levels of groups, such as female/male, child/adolescent/adult, different geographical regions, malnourished/well-nourished, and type of disease agents (viruses, bacteria, protozoa, fungi, helminths, ectoparasites, snakebites). The effects will also be examined to determine how they vary by incentive program design (type, amount, and frequency), implementation plan, study design, and relevant covariates.

### Ethical Considerations

The systematic review protocol has been reviewed and approved by two independent institutional review boards: icddr,b (PR-24112) in Bangladesh and Population Council Institute (IORG0011824) in India. Although systematic reviews do not require institutional review board approvals because no human subjects were involved, these approvals were taken as an institutional requirement.

## Results

**Figure 1.**
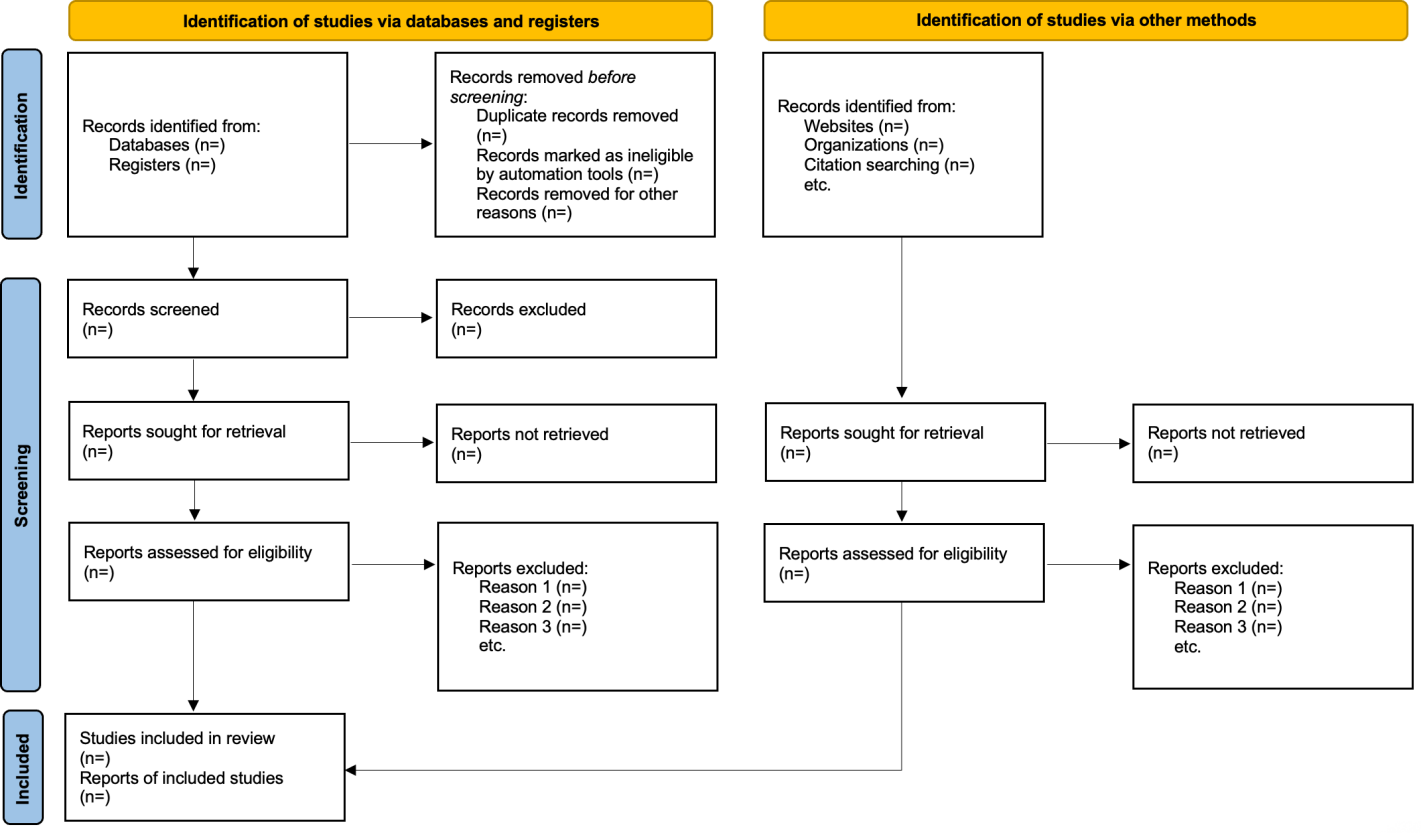
PRISMA (Preferred Reporting Items for Systematic Reviews and Meta-Analyses) flowchart.

This project is supported by the Children’s Investment Fund Foundation and was funded in March 2024. As of January 15, 2026, an exhaustive article search and title-abstract screening have been completed, and the full-text article screening process is expected to be completed by February 2026. Findings will then be synthesized and reported by the end of April 2026. Based on our initial search, we anticipate that 20 to 30 articles will be included, although the final number may vary. The screening process will be reported using the PRISMA (Preferred Reporting Items for Systematic Reviews and Meta-Analyses) flowchart (Figure 1) [55].

## Discussion

### Principal Findings

We expect that this study will be completed by April 2026. This systematic review will critically examine and provide a comprehensive assessment of the effects of cash transfer and other financial incentive programs on NTD-related service utilization and treatment outcomes in LMICs. A recent systematic review of 11 studies published in *The Lancet* found that CCTs improve NTD outcomes through both enhanced living standards from the cash benefits and direct health effects linked to health care–related conditionalities [35]. However, this review had a major limitation in that it included and synthesized evidence for only 3 of the 21 NTD groups prioritized by WHO. In contrast, this review will seek evidence across all 21 WHO-prioritized NTDs.

The financial impact of different categories of financial aid for mitigating NTDs will be assessed to inform policymakers about more effective intervention programs and prevention or treatment strategies. Additionally, the potential link between NTD-related service utilization and health and nutrition outcomes in LMICs will be evaluated. A methodological assessment of the published literature will be performed, and the findings will be compared with those of similar reviews to examine the strength of evidence.

The proposed systematic review will synthesize evidence generated over a 25-year period and will critically assess the strengths and limitations of existing studies on NTDs. The use of rigorous methodology and a robust article screening process is expected to ensure a comprehensive, up-to-date, and unbiased synthesis of evidence on the effectiveness of cash transfer and incentive programs in improving NTD-related service utilization, treatment outcomes, and associated health and nutrition outcomes in LMICs.

One of the major anticipated limitations of this systematic review is that the available evidence may include multiple outcome measures and substantial methodological heterogeneity, potentially limiting the feasibility of meta-analysis. Additional limitations may include potential risk of bias in the included studies, possible publication bias, language bias due to the inclusion of English-language articles only, and limitations of the search strategy. We may face limited sensitivity in the electronic database searches because of the extensive NTD-related keywords and the indexing constraints of certain databases. However, supplementary strategies, including bibliographic citation searching and snowballing from the reference lists of included articles and relevant systematic reviews, are expected to help mitigate these limitations.

### Conclusion

This systematic review will provide policymakers with evidence to support more effective strategic planning and targeted interventions for controlling NTDs in LMICs. The findings from this systematic review will also be valuable for researchers, funding agencies, and governmental bodies seeking to design and implement effective NTD control programs.

### Dissemination Plan

The findings of this systematic review will be presented at national and international conferences and reported in a peer-reviewed journal in accordance with the PRISMA 2020 guideline for reporting systematic reviews [55]. Any amendments to this protocol during the review process will be documented in PROSPERO and reported in the final manuscript.

## Supplementary material

10.2196/76450 Checklist 1 PRISMA-P checklist.

## Abbreviations

CASP: Critical Appraisal Skills Programme
CCT: conditional cash transfer
GRADE: Grading of Recommendations, Assessment, Development and Evaluation
JBI: Joanna Briggs Institute
LMICs: low- and middle-income countries
NTD: neglected tropical disease
PICOS: Population, Interventions, Comparators, Outcomes, Study Design
PRISMA: Preferred Reporting Items for Systematic Reviews and Meta-Analyses
PRISMA-P: Preferred Reporting Items for Systematic Review and Meta-Analysis Protocols
RoB 2: Revised Cochrane Risk of Bias for randomized trials
ROBINS-I: Risk of Bias in Non-Randomized Studies of Interventions
WHO: World Health Organization

## References

[1] Hudu SA, Jimoh AO, Adeshina KA, Otalike EG, Tahir A, Hegazy AA (2024). An insight into the success, challenges, and future perspectives of eliminating neglected tropical disease. Scientific African.

[2] (2024). Global report on neglected tropical diseases 2024. https://iris.who.int/server/api/core/bitstreams/cb44ed69-02bd-4273-b599-26661646f5d3/content.

[3] (2020). Ending the neglect to attain the sustainable development goals: a road map for neglected tropical diseases 2021–2030. https://www.who.int/publications/i/item/9789240010352.

[4] Engels D, Zhou XN (2020). Neglected tropical diseases: an effective global response to local poverty-related disease priorities. Infect Dis Poverty.

[5] Hotez PJ, Fenwick A, Savioli L, Molyneux DH (2009). Rescuing the bottom billion through control of neglected tropical diseases. Lancet.

[6] (2011). The Causes and Impacts of Neglected Tropical and Zoonotic Diseases: Opportunities for Integrated Intervention Strategies.

[7] Coady D (2003). Alleviating structural poverty in developing countries: the approach of PROGRESA in Mexico. https://documents1.worldbank.org/curated/en/435991468757217796/pdf/269430Coady.pdf.

[8] (2017). Integrating neglected tropical diseases into global health and development: fourth WHO report on neglected tropical diseases. https://iris.who.int/server/api/core/bitstreams/903594da-27f0-44f1-a1e8-f4261ed810a5/content.

[9] Fürst T, Salari P, Llamas LM, Steinmann P, Fitzpatrick C, Tediosi F (2017). Global health policy and neglected tropical diseases: then, now, and in the years to come. PLoS Negl Trop Dis.

[10] Patikorn C, Cho JY, Higashi J, Huang XX, Chaiyakunapruk N (2024). Financial hardship among patients suffering from neglected tropical diseases: a systematic review and meta-analysis of global literature. PLOS Negl Trop Dis.

[11] Wagstaff A (2008). Measuring financial protection in health. https://hdl.handle.net/10986/6570.

[12] Nguyen HA, Ahmed S, Turner HC (2023). Overview of the main methods used for estimating catastrophic health expenditure. Cost Eff Resour Alloc.

[13] Adhikari SR, Maskay NM, Sharma BP (2009). Paying for hospital-based care of Kala-azar in Nepal: assessing catastrophic, impoverishment and economic consequences. Health Policy Plan.

[14] Immurana M, Kisseih KG, Abdullahi I (2024). The effects of selected neglected tropical diseases on economic performance at the macrolevel in Africa. BMC Infect Dis.

[15] Magalhães AR, Codeço CT, Svenning JC, Escobar LE, Van de Vuurst P, Gonçalves-Souza T (2023). Neglected tropical diseases risk correlates with poverty and early ecosystem destruction. Infect Dis Poverty.

[16] Tickell KD, Walson JL (2016). Nutritional enteric failure: neglected tropical diseases and childhood stunting. PLoS Negl Trop Dis.

[17] Ngui R, Lim YAL, Chong Kin L, Sek Chuen C, Jaffar S (2012). Association between anaemia, iron deficiency anaemia, neglected parasitic infections and socioeconomic factors in rural children of West Malaysia. PLoS Negl Trop Dis.

[18] Hall A, Zhang Y, Macarthur C, Baker S (2012). The role of nutrition in integrated programs to control neglected tropical diseases. BMC Med.

[19] Quihui-Cota L, Valencia ME, Crompton DWT (2004). Prevalence and intensity of intestinal parasitic infections in relation to nutritional status in Mexican schoolchildren. Trans R Soc Trop Med Hyg.

[20] Pereira PCM (2003). Interaction between infection, nutrition and immunity in tropical medicine. J Venom Anim Toxins incl Trop Dis.

[21] Yap P, Utzinger J, Hattendorf J, Steinmann P (2014). Influence of nutrition on infection and re-infection with soil-transmitted helminths: a systematic review. Parasit Vectors.

[22] Anyolitho MK, Nyakato VN, Huyse T, Poels K, Masquillier C (2023). Health-seeking behaviour regarding schistosomiasis treatment in the absence of a mass drug administration (MDA) program: the case of endemic communities along Lake Albert in Western Uganda. BMC Public Health.

[23] Ackley C, Elsheikh M, Zaman S (2021). Scoping review of neglected tropical disease interventions and health promotion: a framework for successful NTD interventions as evidenced by the literature. PLoS Negl Trop Dis.

[24] Johnston EA, Teague J, Graham JP (2015). Challenges and opportunities associated with neglected tropical disease and water, sanitation and hygiene intersectoral integration programs. BMC Public Health.

[25] Aamar H, Siddiqui JA, Siddiqui A, Essar MY (2023). Neglected tropical diseases in Pakistan: challenges, efforts, and recommendations. Int J Surg.

[26] Engels D, Elphick-Pooley T (2022). The potential of conditional cash transfers for the control of neglected tropical diseases. Lancet Glob Health.

[27] Ramos AN, Heukelbach J, Oliveira M (2020). A conditional cash transfer programme in Brazil improves leprosy treatment outcomes. Lancet Infect Dis.

[28] Novignon J, Prencipe L, Molotsky A (2022). The impact of unconditional cash transfers on morbidity and health-seeking behaviour in Africa: evidence from Ghana, Malawi, Zambia and Zimbabwe. Health Policy Plan.

[29] Evans DK, Holtemeyer B, Kosec K (2019). Cash transfers and health: evidence from Tanzania. World Bank Econ Rev.

[30] Gaarder MM, Glassman A, Todd JE (2010). Conditional cash transfers and health: unpacking the causal chain. J Dev Effect.

[31] Fiszbein A, Schady N, Ferreira FHG (2009). Conditional cash transfers: reducing present and future poverty. https://documents1.worldbank.org/curated/en/914561468314712643/pdf/476030PUB0Cond101Official0Use0Only1.pdf.

[32] Álvarez-Hernández DA, Rivero-Zambrano L, Martínez-Juárez LA, García-Rodríguez-Arana R (2020). Overcoming the global burden of neglected tropical diseases. Ther Adv Infect Dis.

[33] Fernald LCH, Gertler PJ, Neufeld LM (2008). Role of cash in conditional cash transfer programmes for child health, growth, and development: an analysis of Mexico’s Oportunidades. Lancet.

[34] Glassman A, Duran D, Fleisher L (2013). Impact of conditional cash transfers on maternal and newborn health. J Health Popul Nutr.

[35] Ahmed A, Aune D, Vineis P, Pescarini JM, Millett C, Hone T (2022). The effect of conditional cash transfers on the control of neglected tropical disease: a systematic review. Lancet Glob Health.

[36] Hadi A (2001). Promoting health knowledge through micro-credit programmes: experience of BRAC in Bangladesh. Health Promot Int.

[37] McFadden A, Green JM, Williams V (2014). Can food vouchers improve nutrition and reduce health inequalities in low-income mothers and young children: a multi-method evaluation of the experiences of beneficiaries and practitioners of the Healthy Start programme in England. BMC Public Health.

[38] Moher D, Shamseer L, Clarke M (2015). Preferred reporting items for systematic review and meta-analysis protocols (PRISMA-P) 2015 statement. Syst Rev.

[39] Tacconelli E (2010). Systematic reviews: CRD’s guidance for undertaking reviews in health care. Lancet Infect Dis.

[40] Baird S, Ferreira FHG, Özler B, Woolcock M (2013). Relative effectiveness of conditional and unconditional cash transfers for schooling outcomes in developing countries: a systematic review. Campbell Syst Rev.

[41] Banerjee A, Karlan D, Zinman J (2015). Six randomized evaluations of microcredit: introduction and further steps. Am Econ J Appl Econ.

[42] Cunha JM, De Giorgi G, Jayachandran S (2019). The price effects of cash versus in-kind transfers. Rev Econ Stud.

[43] Hoefle-Bénard J, Salloch S (2024). Mass drug administration for neglected tropical disease control and elimination: a systematic review of ethical reasons. BMJ Glob Health.

[44] Mitra AK, Mawson AR (2017). Neglected tropical diseases: epidemiology and global burden. Trop Med Infect Dis.

[45] (2018). WHO to publish first official guidelines on leprosy diagnosis, treatment and prevention [internet]. World Health Organization.

[46] Sharma P, Mukherjee R, Talwar GP (2005). Immunoprophylactic effects of the anti-leprosy Mw vaccine in household contacts of leprosy patients: clinical field trials with a follow up of 8-10 years. Lepr Rev.

[47] Lwin K, Sundaresan T, Gyi MM (1985). BCG vaccination of children against leprosy: fourteen-year findings of the trial in Burma. Bull World Health Organ.

[48] Macfarlane CL, Budhathoki SS, Johnson S, Richardson M, Garner P (2019). Albendazole alone or in combination with microfilaricidal drugs for lymphatic filariasis. Cochrane Database Syst Rev.

[49] Ouzzani M, Hammady H, Fedorowicz Z, Elmagarmid A (2016). Rayyan-a web and mobile app for systematic reviews. Syst Rev.

[50] Sterne JAC, Savović J, Page MJ (2019). RoB 2: a revised tool for assessing risk of bias in randomised trials. BMJ.

[51] Sterne JA, Hernán MA, Reeves BC (2016). ROBINS-I: a tool for assessing risk of bias in non-randomised studies of interventions. BMJ.

[52] (2018). CASP checklist: CASP qualitative studies checklist. CASP.

[53] Jacobs W, Downey LE (2022). Impact of conditional cash transfer programmes on antenatal care service uptake in low and middle-income countries: a systematic review. BMJ Open.

[54] Hunter BM, Harrison S, Portela A, Bick D (2017). The effects of cash transfers and vouchers on the use and quality of maternity care services: a systematic review. PLoS One.

[55] Page MJ, McKenzie JE, Bossuyt PM (2021). The PRISMA 2020 statement: an updated guideline for reporting systematic reviews. Syst Rev.

