# Supplementary material for: Effects of Cash Transfer and Incentive Programs on Service Utilization and Treatment Outcomes Related to Neglected Tropical Diseases and Their Impact on Health and Nutrition in Low- and Middle-Income Countries: Protocol for a Systematic Review
Source: JMIR Res Protoc. 2026 Mar 19;15:e76450. doi: 10.2196/76450 (PMC13006489; doi:10.2196/76450)
Supplement: Checklist 1 [file resprot-v15-e76450-s001.pdf]

PRISMA-P 2015 Checklist

Section/topic	#	Checklist item	Information reported		Line number(s)
			Yes	No	
ADMINISTRATIVE INFORMATION					
Title					
Identification	1a	Identify the report as a protocol of a systematic review	☒	☐	33<br>144
Update	1b	If the protocol is for an update of a previous systematic review, identify as such	☐	☒	-
Registration	2	If registered, provide the name of the registry (e.g., PROSPERO) and registration number in the Abstract	☐	☒	72<br>164
Authors					
Contact	3a	Provide name, institutional affiliation, and e-mail address of all protocol authors; provide physical mailing address of corresponding author	☒	☐	6-24
Contributions	3b	Describe contributions of protocol authors and identify the guarantor of the review	☒	☐	444-448
Amendments	4	If the protocol represents an amendment of a previously completed or published protocol, identify as such and list changes; otherwise, state plan for documenting important protocol amendments	☐	☒	-
Support					
Sources	5a	Indicate sources of financial or other support for the review	☒	☐	436-439
Sponsor	5b	Provide name for the review funder and/or sponsor	☒	☐	437
Role of sponsor/funder	5c	Describe roles of funder(s), sponsor(s), and/or institution(s), if any, in developing the protocol	☒	☐	437-438
INTRODUCTION					
Rationale	6	Describe the rationale for the review in the context of what is already known	☒	☐	134-144
Objectives	7	Provide an explicit statement of the question(s) the review will address with reference to participants, interventions, comparators, and outcomes (PICO)	☒	☐	153-158

Section/topic	#	Checklist item	Information reported		Line number(s)
			Yes	No	
METHODS					
Eligibility criteria	8	Specify the study characteristics (e.g., PICO, study design, setting, time frame) and report characteristics (e.g., years considered, language, publication status) to be used as criteria for eligibility for the review	☒	☐	160-216
Information sources	9	Describe all intended information sources (e.g., electronic databases, contact with study authors, trial registers, or other grey literature sources) with planned dates of coverage	☒	☐	250-263
Search strategy	10	Present draft of search strategy to be used for at least one electronic database, including planned limits, such that it could be repeated	☒	☐	247
STUDY RECORDS					
Data management	11a	Describe the mechanism(s) that will be used to manage records and data throughout the review	☒	☐	265-267
Selection process	11b	State the process that will be used for selecting studies (e.g., two independent reviewers) through each phase of the review (i.e., screening, eligibility, and inclusion in meta-analysis)	☒	☐	270-283
Data collection process	11c	Describe planned method of extracting data from reports (e.g., piloting forms, done independently, in duplicate), any processes for obtaining and confirming data from investigators	☒	☐	286-297
Data items	12	List and define all variables for which data will be sought (e.g., PICO items, funding sources), any pre-planned data assumptions and simplifications	☒	☐	166-297
Outcomes and prioritization	13	List and define all outcomes for which data will be sought, including prioritization of main and additional outcomes, with rationale	☒	☐	198-216
Risk of bias in individual studies	14	Describe anticipated methods for assessing risk of bias of individual studies, including whether this will be done at the outcome or study level, or both; state how this information will be used in data synthesis	☒	☐	299-307
DATA					
Synthesis	15a	Describe criteria under which study data will be quantitatively synthesized	☒	☐	315-357

Section/topic	#	Checklist item	Information reported		Line number(s)
			Yes	No	
	15b	If data are appropriate for quantitative synthesis, describe planned summary measures, methods of handling data, and methods of combining data from studies, including any planned exploration of consistency (e.g., I 2, Kendall's tau)	☒	☐	315-357
	15c	Describe any proposed additional analyses (e.g., sensitivity or subgroup analyses, meta-regression)	☒	☐	359-367
	15d	If quantitative synthesis is not appropriate, describe the type of summary planned	☒	☐	315-357
Meta-bias(es)	16	Specify any planned assessment of meta-bias(es) (e.g., publication bias across studies, selective reporting within studies)	☒	☐	413-422
Confidence in cumulative evidence	17	Describe how the strength of the body of evidence will be assessed (e.g., GRADE)	☒	☐	335-338
